# Supplementary material for: Proximity extracellular protein-protein interaction analysis of EGFR using AirID-conjugated fragment of antigen binding
Source: Nat Commun. 2023 Dec 14;14:8301. doi: 10.1038/s41467-023-43931-7 (PMC10721602; doi:10.1038/s41467-023-43931-7)
Supplement: Supplementary file 1 — Supplementary Information [file 41467_2023_43931_MOESM1_ESM.pdf]

Supplementary Information

Proximity extracellular protein-protein interaction analysis of EGFR using AirID-conjugated fragment of antigen binding

Kohdai Yamada[1], Ryouhei Shioya[1], Kohei Nishino[2], Hirotake Furihata[1], Atsushi Hijikata[3], Mika K Kaneko[4,5], Yukinari Kato[4,5], Tsuyoshi Shirai[6], Hidetaka Kosako[2*], and Tatsuya Sawasaki[1*]

[1] *Division of Cell-Free Life Science, Proteo-Science Center, Ehime University, 3 Bunkyo-cho, Matsuyama, Ehime 790-8577, Japan.*

[2]*Division of Cell Signaling, Fujii Memorial Institute of Medical Sciences, Institute of Advanced Medical Sciences, Tokushima University, Tokushima 770-8503, Japan.*

[3]*Laboratory of Computational Genomics, School of Life Sciences, Tokyo University of Pharmacy and Life Sciences, Hachioji, 192-0392, Japan.*

[4]*Department of Antibody Drug Development, Tohoku University Graduate School of Medicine, Sendai, 980-8575, Japan.*

[5]*Department of Molecular Pharmacology, Tohoku University Graduate School of Medicine, Sendai, 980-8575, Japan.*

[6]*Department of Bioscience, Nagahama Institute of BioScience and Technology, 1266 Tamura, Nagahama, 526-0829, Japan.*

* Correspondence to: sawasaki@ehime-u.ac.jp; kosako@tokushima-u.ac.jp.

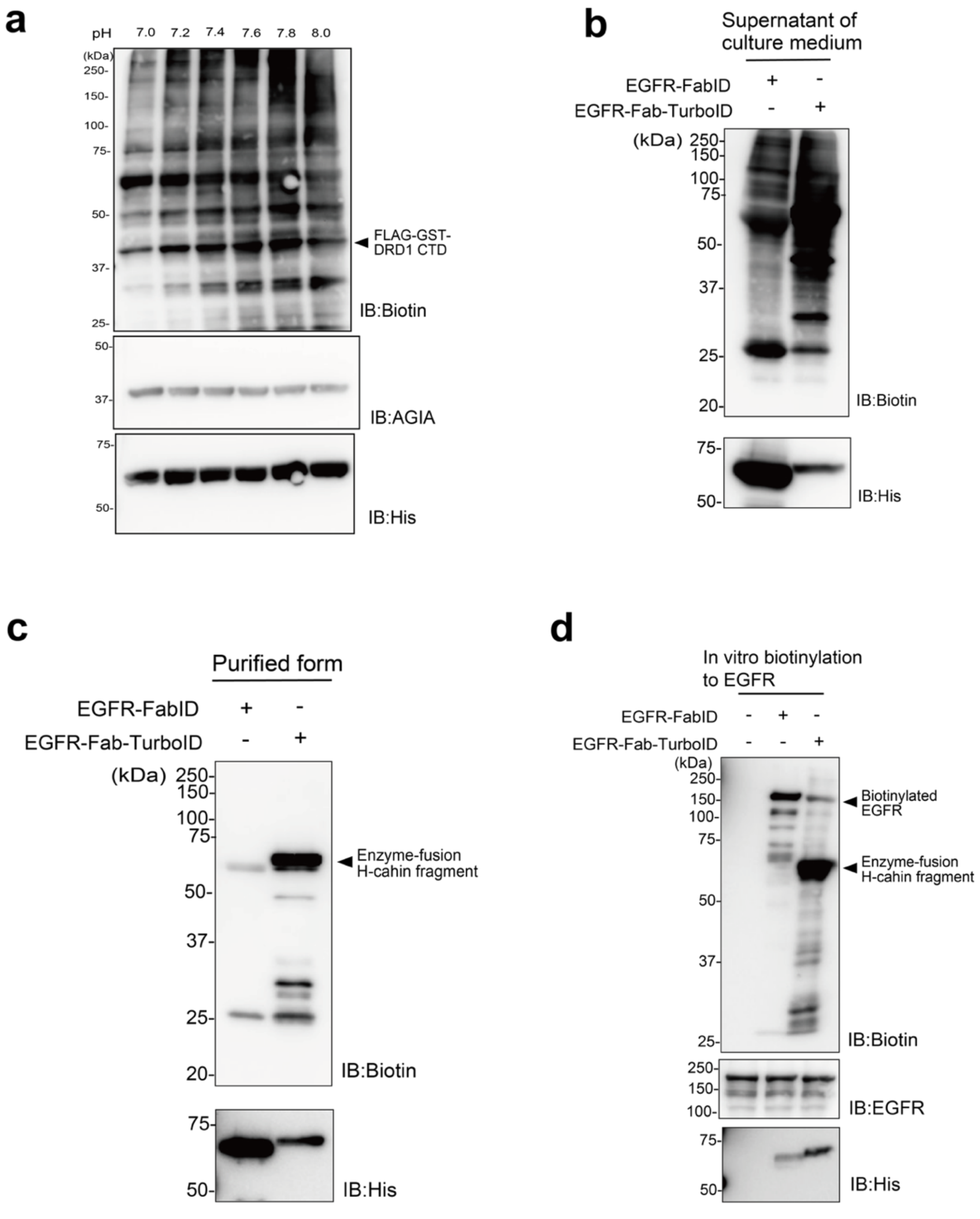

Supplementary Fig. 1. Evaluation of biotinylation activity by pH of AGIA-FabID and comparison of EGFR-FabID with TurboID-fused EGFR-Fab.

a, *In vitro* biotinylation of AGIA-FabID to antigen in the pH range of 7.0 to 8.0. **b,** Evaluation of

biotinylation of EGFR-FabID and EGFR-Fab-TurboID synthesised in Expi293F cells on exposure to the supernatant of culture medium. Cultures were grown for one week after transfection with plasmids. **c,** Evaluation of self-biotinylation of purified EGFR-FabID and EGFR-Fab-TurboID by immunoblotting using an anti-biotin antibody. **d,** Biotinylation assay using EGFR-FabID and EGFR-Fab-TurboID *in vitro*. EGFR was synthesised using a wheat cell-free protein synthesis system-based Disulfide Bond PLUS Expression Kit. Source data are provided as a Source Data file.

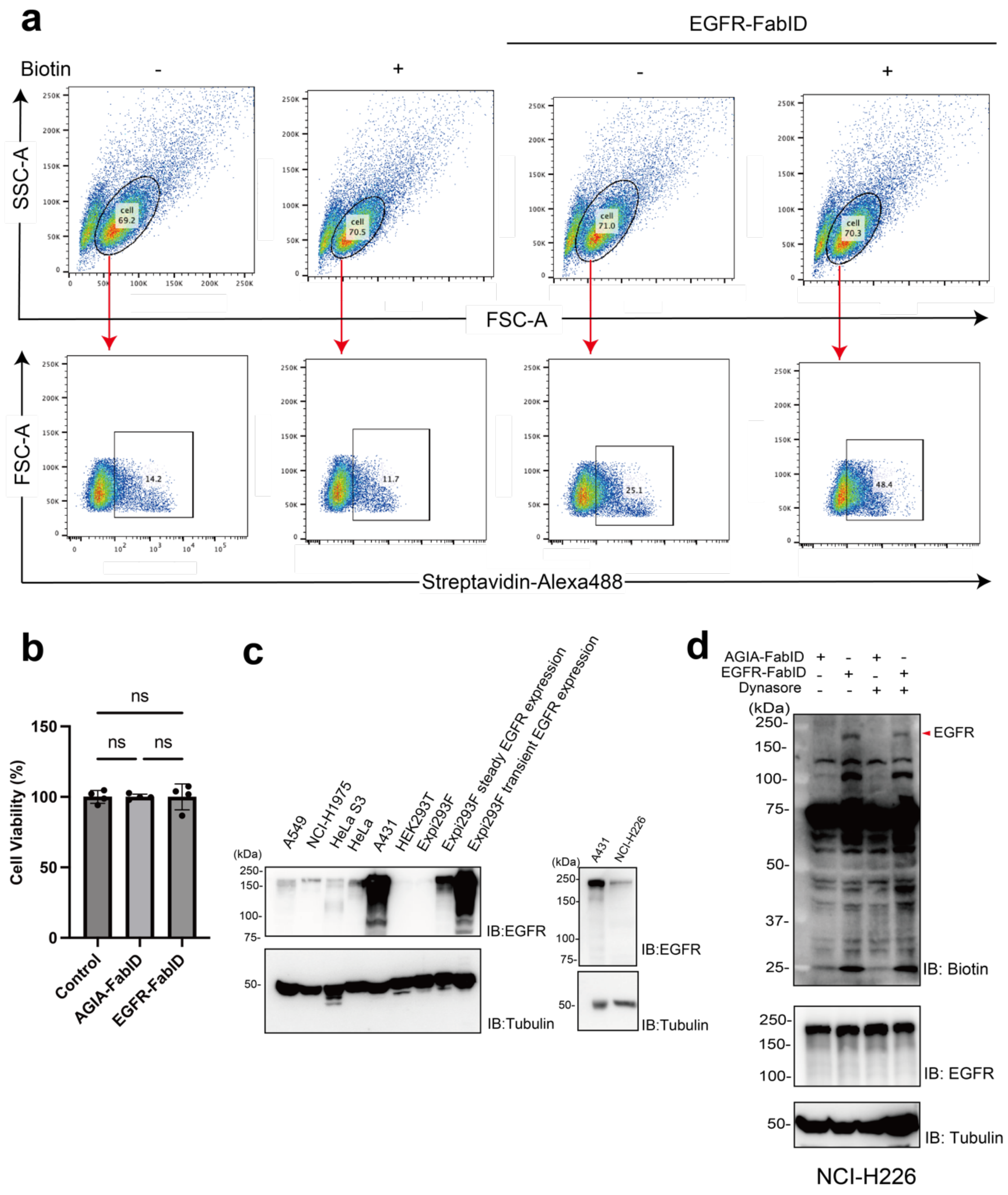

Supplementary Fig. 2. Flow cytometry to confirm cell surface biotinylation of EGFR-FabID and to measurement of cytotoxicity of FabID.

a, EGFR-FabID-mediated cell surface biotinylation of Expi293F cells stably expressing EGFR biotinylated by EGFR-FabID was observed by flow cytometry. Dead cells were excluded based on

FSC-A and SSC-A characteristics. Fluorescence was measured after staining with Alexa Fluor 488-conjugated streptavidin. **b,** Measurement of FabID cytotoxicity using the MTS assay. A431 cells were treated with EGFR-FabID, AGIA-FabID, or PBS (control) for 2 h before performing the MTS assay. No significant differences were detected by one-way ANOVA ($P < 0.001$). Error bars represent standard deviations. **c,** Immunoblot analysis of EGFR expression in various cells.

d, EGFR-FabID-mediated biotinylation of EGFR after the addition of the endocytosis inhibitor Dynasore. NCI-H226 cells were used for biotinylation with EGFR-FabID after 1 h of Dynasore addition. Source data are provided as a Source Data file.

a

			A431 Endogenous EGFR				293F Transietnly over-expression EGFR	293F EGFR stable expression
			Control	EGF	Gefitinib	EGF + Gefitinib	Normal condition	Normal condition
	Biotinylation site (Lys)	Distance betweenn Ser380 and biotinylation site (Å)	Fold increase*	Fold increase*	Fold increase*	Fold increase*	Fold increase*	Fold increase*
Extracellular	133	45.3	ND	ND	ND	ND	4.3 ± 2.4	ND
	293 or 294	54.9	0.9 ± 0.2	0.5 ± 0.0	ND	0.5 ± 0.1	ND	ND
	335	48.0	0.8 ± 0.3	0.6 ± 0.0	ND	0.5 ± 0.2	ND	ND
	487 or 489	42.1 or 31.4	12.4 ± 1.3	14.7 ± 1.6	25.1 ± 2.8	46.0 ± 6.5	79.1 ± 5.7	14.9 ± 2.2
	479	44.6	3.4 ± 0.8	5.0 ± 0.4	5.1 ± 0.7	14.5 ± 1.0	40.8 ± 4.9	ND
	478	44.0	0.7 ± 0.1	0.6 ± 0.1	ND	0.7 ± 0.2	ND	ND
	454	44.5	1.9 ± 0.1	2.1 ± 0.2	2.4 ± 0.1	2.0 ± 0.8	11.6 ± 1.5	ND
	489	31.4	0.8 ± 0.2	3.1 ± 0.2	ND	3.3 ± 0.2	2.4 ± 0.3	1.5 ± 0.2
	479 or 487 or 489	44.6 or 42.1 or 31.4	ND	ND	14.1 ± 1.8	ND	ND	ND

* EGFR-FabID / AGIA-FabID

b

		293F Transietnly over-expression EGFR	293F EGFR stable expression	A431 Endogenous EGFR		
		Normal condition	Normal condition	Control	EGF	EGF + Gefitinib
	Biotinylation site (Lys)	Fold increase*	Fold increase*	Fold increase*	Fold increase*	Fold increase*
Intracellular	716	4.7 ± 1.4	ND	ND	ND	ND
	739	1.8 ± 1.7	ND	ND	ND	ND
	745	2.4 ± 0.2	ND	ND	ND	ND
	754	2.1 ± 0.4	ND	ND	ND	ND
	846	7.0 ± 2.4	ND	ND	ND	ND
	852	5.3 ± 0.5	ND	ND	ND	ND
	1188	9.1 ± 1.5	ND	ND	ND	ND

* EGFR-FabID / AGIA-FabID

c

			NCI-H226 Endogenous EGFR			
			Control	EGF	Gefitinib	EGF + Gefitinib
	Biotinylation site (Lys)	Distance betweenn Ser380 and biotinylation site (Å)	Fold increase*	Fold increase*	Fold increase*	Fold increase*
Extracellular	489	31.4	1.5 ± 0.1	11.5 ± 0.7	ND	5.0 ± 1.0
	487 or 489	42.1 or 31.4	1.9 ± 0.5	8.2 ± 1.4	1.7 ± 0.2	2.1 ± 0.8
	335	36.9	4.4 ± 1.2	5.2 ± 0.8	ND	0.6 ± 0.1
	293 or 294	54.9 or 48.0	1.4 ± 0.2	3.8 ± 1.3	0.9 ± 0.2	1.3 ± 0.4
	226	44.5	2.5 ± 0.7	1.9 ± 0.4	ND	1.8 ± 0.7
	478 or 479	31.4	1.1 ± 0.6	1.5 ± 0.2	ND	2.7 ± 0.3
	489	31.4	ND	ND	1.3 ± 0.2	ND

* EGFR-FabID / AGIA-FabID

Supplementary Fig. 3. Comparison of EGFR biotinylation by EGFR-FabID between Expi293F, A431, and NCI-H226 cells.

a,b,c, Distance of the EGFR biotinylation site from S380 and biotinylation ratio increase in EGFR-FabID compared with AGIA-FabID. Each treatment section containing A431 and Expi293F cells

was biotinylated using EGFR-FabID in three independent replicates. Each section treated with NCI-H226 cells was biotinylated with EGFR-FabID in five independent 10 cm dishes. Cell lysates were treated in independent replicates and pooled. The pooled samples were divided into three samples, purified separately, and analysed by mass spectrometry. The ± in the figure represents the standard deviation.

a

A431

Protein name	Uniprot code	DTX edge
ANXA2	P07355	1
CANX	P27824	1
CFL1	P23528	1
DSG2	Q14126	2
EEF1A1	P68104	1
EFNB1	P98172	1
EGFR	P00533	0
ENO1	P06733	2
GAPDH	P04406	1
HSP90AA1	P07900	1
HSP90AB1	P08238	1
HSPA8	P11142	1
ICAM1	P05362	1
MAPT	P10636	1
SLC3A2	P08195	1
ADAM17	P78536	2
ALPP	P05187	3
CA2	P00918	3
CD44	P16070	2
CDH3	P22223	2
CDH5	P33151	2
CKMT1B	P12532	2
DCBLD2	Q96PD2	2
DSG3	P32926	999
EPCAM	P16422	3
F11R	Q9Y624	2
GPC1	P35052	2
HLA-A	P04439	1
HLA-B	P01889	2
HLA-C	P10321	2
INSR	P06213	2
ITGA6	P23229	2
LAMA3	Q16787	3
LAMB3	Q13751	2
MICA	Q29983	2
NEO1	Q92859	3
PCDH1	Q08174	3
PLXNB2	O15031	2
PPIB	P23284	2
PTK7	Q13308	2
RUVBL1	Q9Y265	2
ST14	Q9Y5Y6	2
TACSTD2	P09758	3
TGFBI	Q15582	3

NCI-H226

Protein name	Uniprot code	DTX edge
ALCAM	Q13740	1
ANXA2	P07355	1
ATP5F1A	P25705	1
DSG2	Q14126	2
EEF1A1	P68104	1
EFNB1	P98172	1
EGFR	P00533	0
ENO1	P06733	2
EZR	P15311	1
GAPDH	P04406	1
ICAM1	P05362	1
ITGB4	P16144	1
KRT1	P04264	1
LRP1	Q07954	1
PTPRF	P10586	1
SLC3A2	P08195	1
ADAM17	P78536	2
ADGRG6	Q86SQ4	3
AGRN	O00468	2
ANOS1	P23352	3
ARFGAP2	Q8N6H7	2
ATP1B3	P54709	2
BCAM	P50895	2
BSG	P35613	2
CA2	P00918	3
CD44	P16070	2
CD99L2	Q8TCZ2	3
CDH11	P55287	2
CEMIP	Q8WUJ3	2
CEMIP2	Q9UHN6	2
CLINT1	Q14677	2
CLU	P10909	2
COL18A1	P39060	2
DCBLD2	Q96PD2	2
DSC3	Q14574	2
EFNA5	P52803	2
EPHB6	O15197	2
FAS	P25445	2
FGFBP1	Q14512	3
GABARAP	O95166	2
GPC1	P35052	2
HLA-A	P04439	1
HLA-C	P10321	2
HSPG2	P98160	2
INSR	P06213	2
ITGA3	P26006	2
ITGA6	P23229	2
ITGB1	P05556	2
KHDRBS1	Q07666	2
L1CAM	P32004	4
LAMA5	O15230	2
MELTF	P08582	3
MICA	Q29983	2
NFASC	O94856	2
OLR1	P78380	2
PLXNB2	O15031	2
PODXL	O00592	2
PTK7	Q13308	2
S100A10	P60903	2
SEPTIN2	Q15019	2
SLC39A10	Q9ULF5	3
SQSTM1	Q13501	2
STXBP1	P61764	2
TENM2	Q9NT68	2
TFRC	P02786	2
VASN	Q6EMK4	3

b

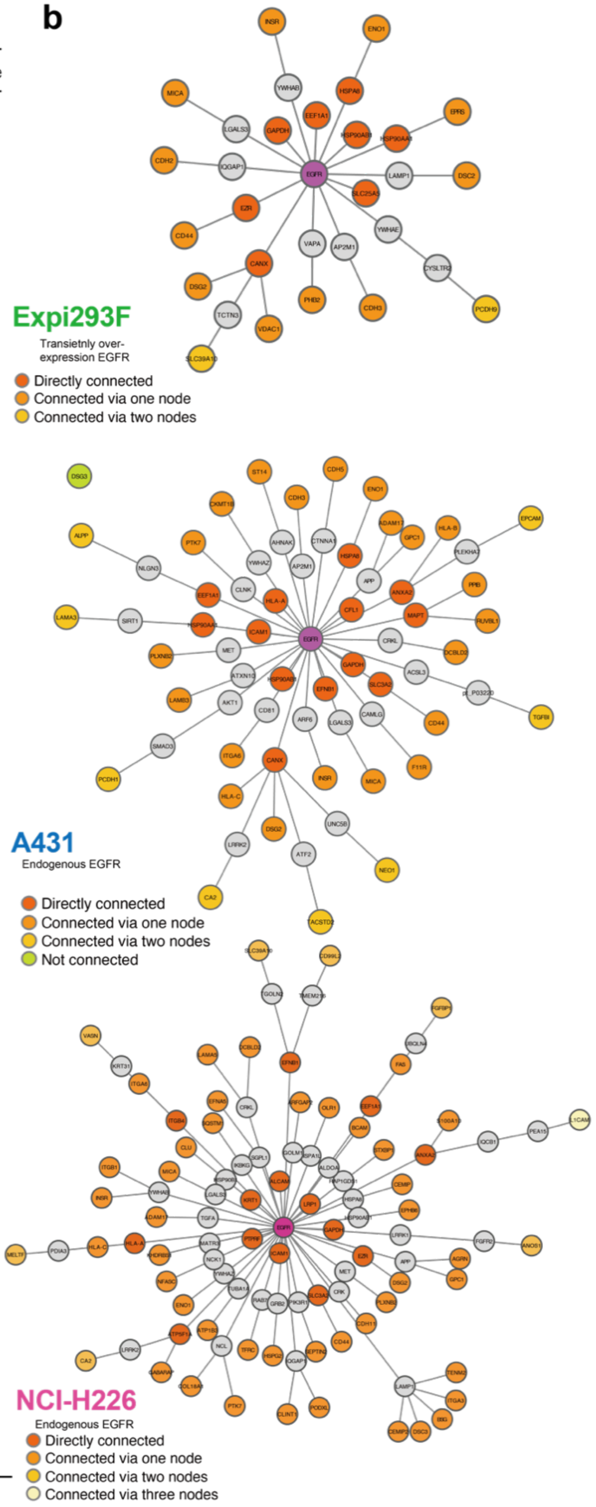

Supplementary Fig. 4. Proximal exPPI networks of EGFR in each cell line.

a, List of cell surface proteins predominantly biotinylated by EGFR-FabID in A431 and NCI-H226 cells. The number of DTX edges for each protein and EGFR are shown in the table (EGFR-FabID/AGIA-FabID ratios > 1 and $P < 0.05$). **b,** Pathway diagram of cell surface proteins identified by EGFR-FabID in each cell type. Proteins in grey were not detected by biotinylation in this study but relayed cell surface protein-protein interactions.

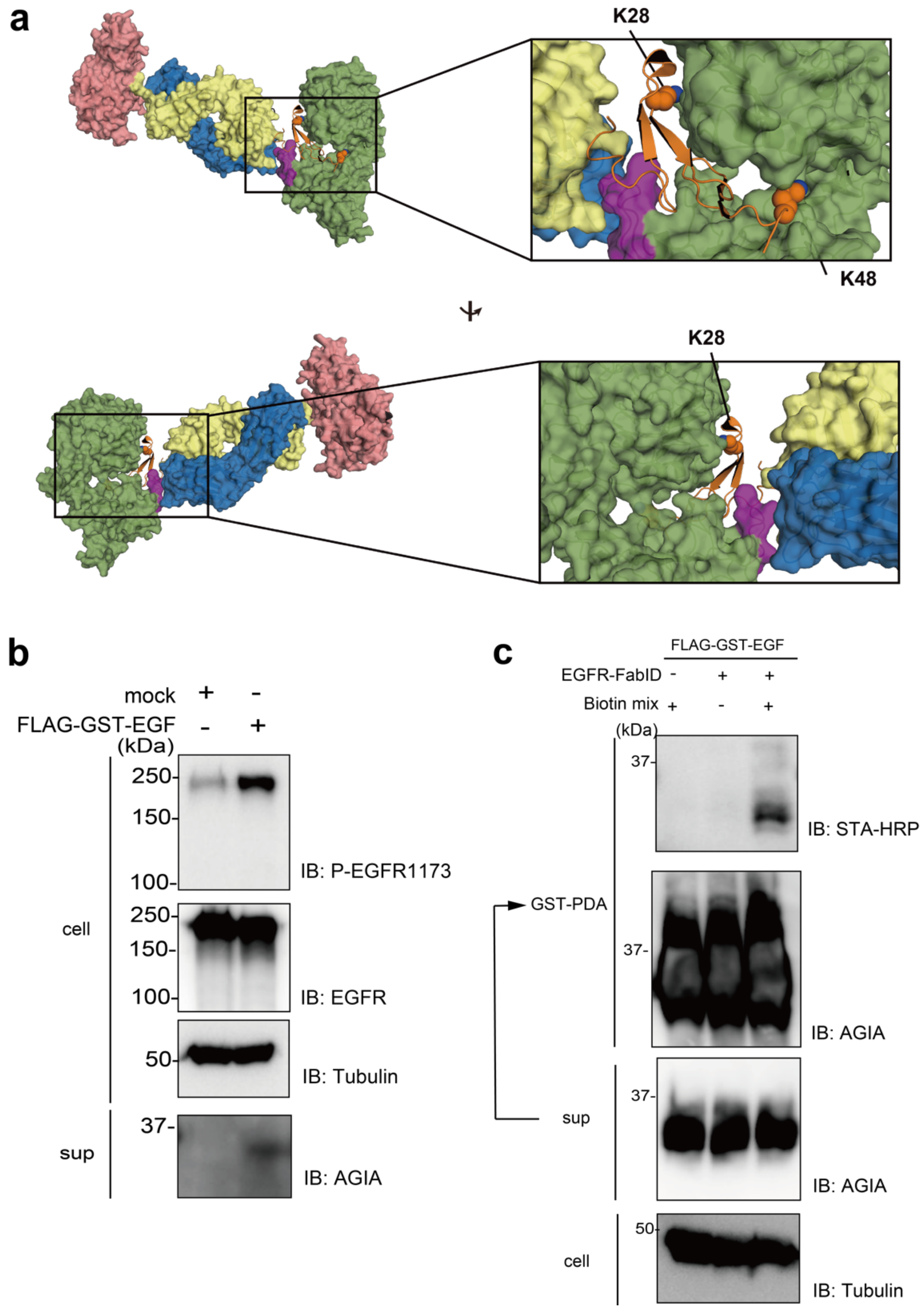

Supplementary Fig. 5. Diagram of the EGFR, EGF, and EGFR-FabID ternary complex model.

a, EGFR-FabID, EGFR, and EGF modelling diagrams were constructed from the structural information and AlphaFold. The structure of the EGFR-EGF complex was obtained from PDB:1IVO. Green and purple represents EGFR, orange represents EGF, and yellow and blue represent the anti-EGFR Fab antibodies. Pink represents AirID. All lysine residues (K28 and K48) of EGF are shown. **b,** Kinase assay with FLAG-GST-EGF (FLAG-GST-TEV-His-AGIA-GS linker-EGF) overexpressed in Expi293F cells stably expressing EGFR. The vector from pcDNA3.1 was transfected for mock and pcDNA3.4-FLAG-GST-TEV-His-AGIA-GS linker-EGF for FLAG-GST-EGF. After 24 hours of transfection, enhancer of Expi293F Transfection Kit was added. Cells and culture medium were recovered 4 hours after enhancer addition. Phosphorylation of EGFR induced by FLAG-GST-EGF was then confirmed by immunoblotting. Sup represents culture medium and cell represents cell lysate. **c,** GST pull-down assay using biotinylated cultures by EGFR-FabID after overexpression of FLAG-GST-EGF in Expi293F cells stably expressing EGFR. EGFR-FabID was added to the medium and biotinylation reaction was performed for 2 hours. The medium was collected after the biotinylation reaction and GST pull-down was performed using the medium. After GST pull-down, the immunoblotting was performed and biotinylation was identified by STA-HRP. Source data are provided as a Source Data file.

a

A431

Control	EGF	EGF+Gefitinib
Protein name	Protein name	Protein name
ANXA2	ANXA2	DSG2
CANX	CANX	EEF1A1
CFL1	DSG2	EGFR
EEF1A1	EFNB1	ENO1
EGFR	EGFR	GAPDH
ENO1	ENO1	HSP90AA1
HSP90AA1	GAPDH	HSP90AB1
HSP90AB1	HSP90AA1	HSPA8
ADAM17	HSP90AB1	ICAM1
CA2	HSPA8	SLC3A2
CDH3	ICAM1	ADAM17
CDH5	MAPT	CA2
DCBLD2	ADAM17	CD44
DSG3	ALPP	CDH3
EPCAM	CA2	CDH5
F11R	CDH3	CKMT1B
GPC1	CDH5	DCBLD2
HLA-B	CKMT1B	DSG3
HLA-C	DCBLD2	EPCAM
INSR	DSG3	GPC1
ITGA6	EPCAM	HLA-A
LAMB3	GPC1	HLA-B
MICA	HLA-C	HLA-C
NEO1	INSR	INSR
PCDH1	ITGA6	ITGA6
PLXNB2	LAMA3	MICA
PPIB	MICA	NEO1
PTK7	PLXNB2	PLXNB2
RUVBL1	PTK7	PTK7
ST14	ST14	RUVBL1
	TACSTD2	ST14
		TACSTD2
		TGFBI

NCI-H226

Control	EGF	EGF+Gefitinib
Protein name	Protein name	Protein name
EGFR	EGFR	EGFR
ANXA2	ANXA2	ANXA2
DSG2	DSG2	ATP5F1A
EEF1A1	EEF1A1	DSG2
EFNB1	ENO1	EEF1A1
ENO1	GAPDH	EFNB1
EZR	ICAM1	ENO1
GAPDH	KRT1	EZR
ICAM1	LRP1	GAPDH
KRT1	PTPRF	ITGB4
LRP1	ADAM17	LRP1
SLC3A2	ADGRG6	ALCAM
ADGRG6	AGRN	CA2
ANOS1	ATP1B3	CD44
ARFGAP2	BSG	CDH11
ATP1B3	CA2	CEMIP
BCAM	CD99L2	CLU
BSG	CDH11	DSC3
CA2	CEMIP	FAS
CD99L2	CLINT1	GABARAP
CDH11	CLU	GPC1
CEMIP	COL18A1	HLA-A
CEMIP2	DCBLD2	INSR
DCBLD2	DSC3	ITGA3
DSC3	EFNA5	ITGA6
EFNA5	EPHB6	ITGB1
GABARAP	FGFBP1	KHDRBS1
GPC1	GABARAP	MICA
HSPG2	GPC1	PLXNB2
INSR	HLA-A	PODXL
ITGB1	HLA-C	PTK7
KHDRBS1	HSPG2	S100A10
L1CAM	INSR	STXBP1
MELTF	ITGA3	TENM2
MICA	ITGA6	
OLR1	KHDRBS1	
PLXNB2	LAMA5	
PTK7	MELTF	
S100A10	MICA	
SEPTIN2	NFASC	
SLC39A10	PTK7	
STXBP1	S100A10	
VASN	SLC39A10	
	SQSTM1	
	STXBP1	
	TENM2	
	TFRC	
	VASN	

Supplementary Fig. 6. Changes in biotinylated proteins in A431 and NCI-H226 cells on adding EGF or gefitinib.

a, Table of extracellular proteins identified by mass spectrometry (EGFR-FabID/AGIA-FabID ratios > 1, and $P < 0.05$). Proteins in black font are those already known to interact with EGFR, whereas those in blue font represent new EGFR-interacting proteins.

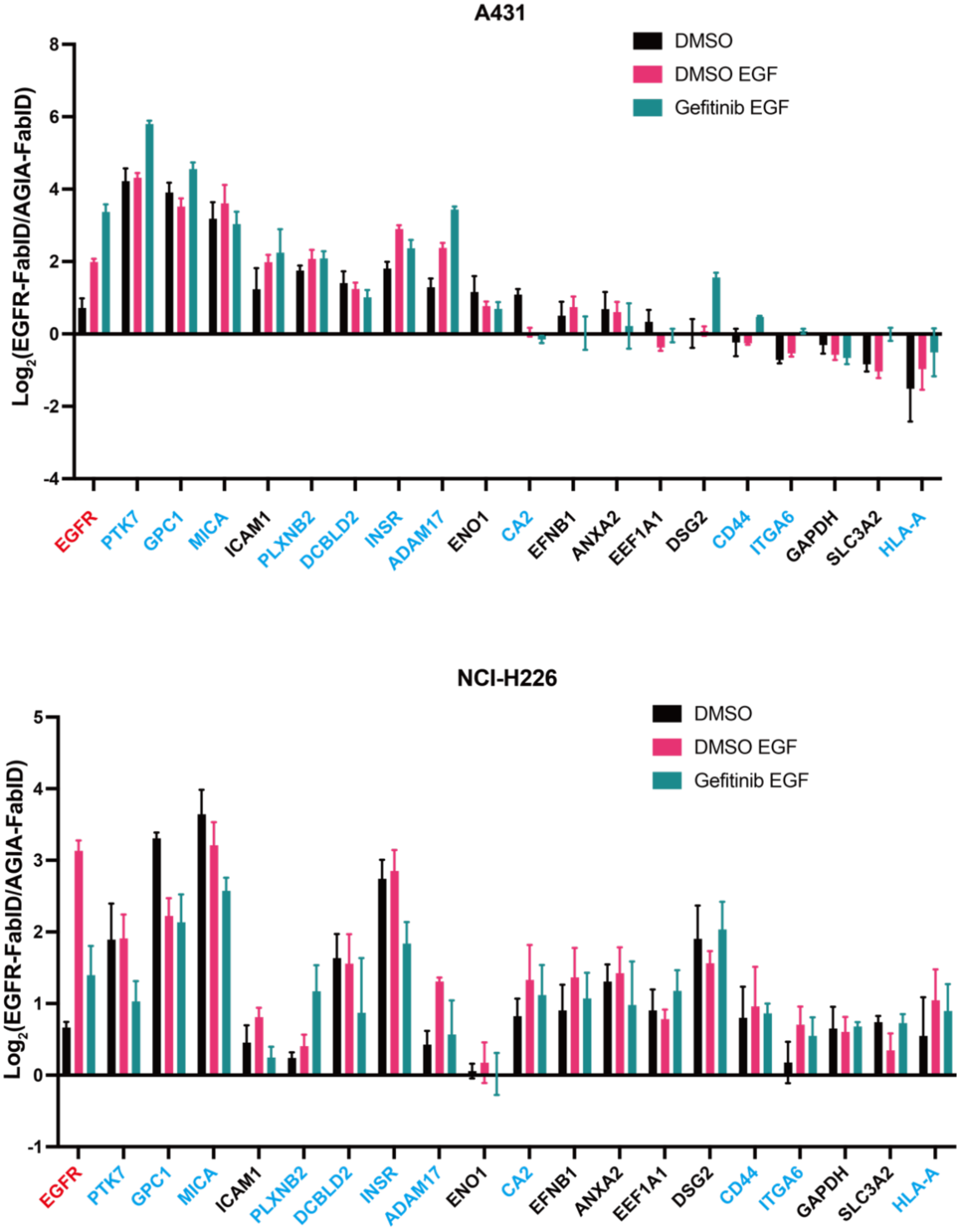

Supplementary Fig. 7. Effects of EGF and Gefitinib on biotinylation by EGFR-FabID in A431 and NCI-H226 cells.

The proteins commonly biotinylated by EGFR-FabID in A431 and NCI-H226 cells were quantified by LC-MS/MS analysis. Error bars represent standard deviations.

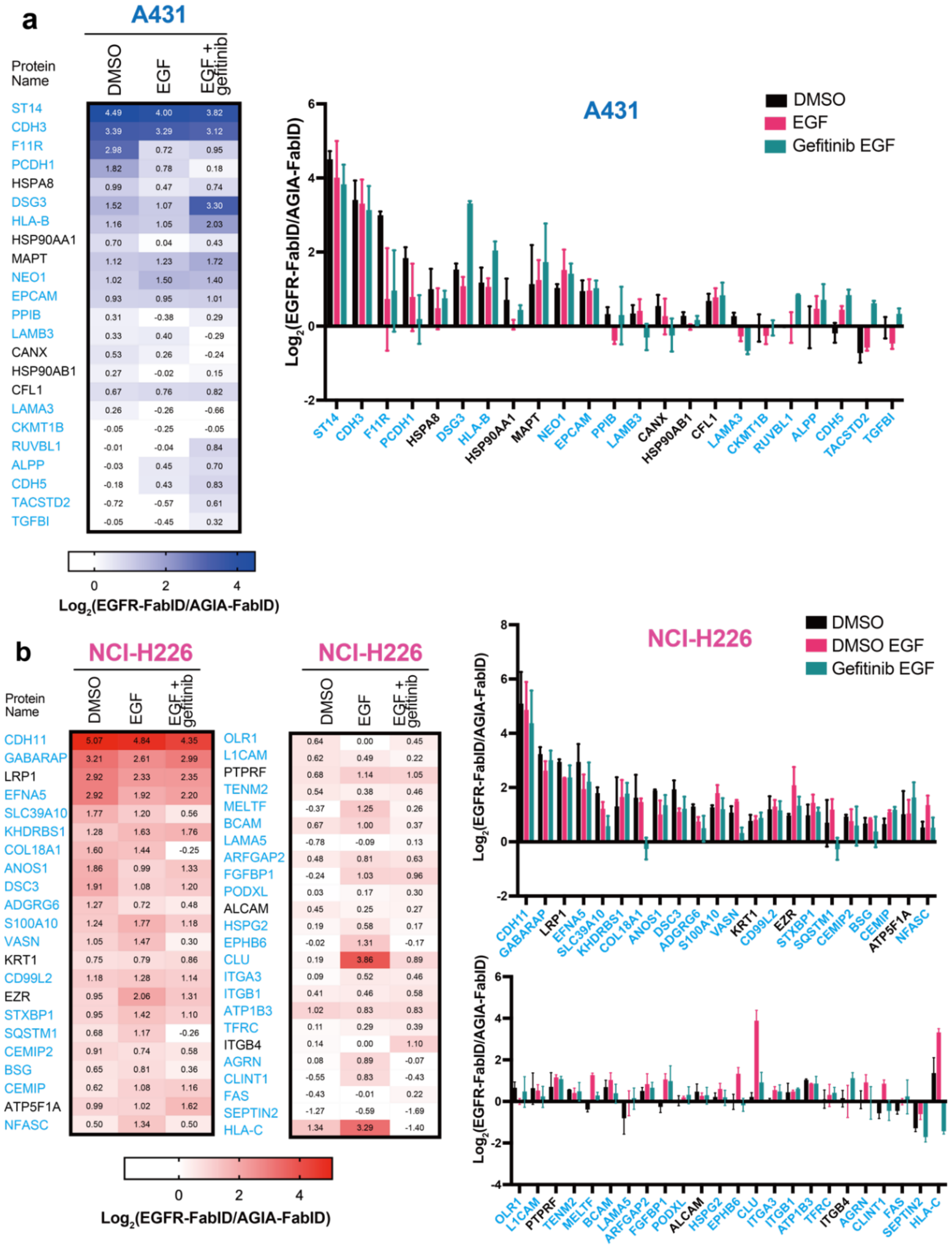

Supplementary Fig. 8. Comparison of biotinylation ratio among control (DMSO), EGF, and EGF + gefitinib in A431 and NCI-H226 cells.

a,b, Heat map showing changes in biotinylation of cell surface proteins upon addition of EGF or gefitinib to A431 cells (a) or NCI-H226 cells (b). Each cell indicates an average value. Bar chart showing quantification of biotinylated proteins in A431 cells by LC-MS/MS. Error bars represent standard deviations.

a

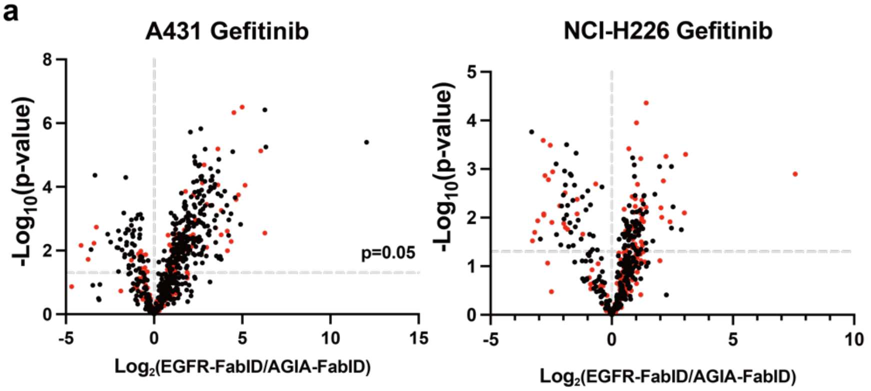

b

A431 Gefitinib

Protein name	Uniprot code	DTX edge
EGFR	P00533	0
ANXA1	P04083	1
ATP5F1B	P06576	1
CANX	P27824	1
CTTN	Q14247	1
DSG2	Q14126	2
EEF1A1	P68104	1
EFNB1	P98172	1
ENO1	P06733	2
GAPDH	P04406	1
HSP90AA1	P07900	1
HSP90AB1	P08238	1
HSPA8	P11142	1
ITGB4	P16144	1
JUP	P14923	1
KRT1	P04264	1
MET	P08581	1
SLC25A5	P05141	1
ADAM17	P78536	2
ADGRF1	Q5T601	999
BSG	P35613	2
CAP1	Q01518	2
CD44	P16070	2
CDH3	P22223	2
CDH5	P33151	2
CELSR1	Q9NYQ6	3
DCBLD2	Q96PD2	2
DSG3	P32926	999
EFNA5	P52803	2
EIF5AL1	Q6IS14	3
EPCAM	P16422	3
F11R	Q9Y624	2
GPC1	P35052	2
HLA-A	P04439	1
HLA-B	P01889	2
INSR	P06213	2
ITGA6	P23229	2
ITGB1	P05556	2
LAMA3	Q16787	3
MICA	Q29983	2
MSN	P26038	2
NECTIN1	Q15223	3
P4HB	P07237	2
PCDH1	Q08174	3
PLXNB2	O15031	2
PPIB	P23284	2
PTK7	Q13308	2
PTPRS	Q13332	2
S100A10	P60903	2
S100A6	P06703	2
ST14	Q9Y5Y6	2
TACSTD2	P09758	3
TGFBI	Q15582	3

NCI-H226 Gefitinib

Protein name	Uniprot code	DTX edge
EGFR	-	-
ALCAM	Q13740	1
ANXA2	P07355	1
CANX	P27824	1
DSG2	Q14126	2
EEF1A1	P68104	1
ENO1	P06733	2
EZR	P15311	1
GAPDH	P04406	1
ICAM1	P05362	1
KRT1	P04264	1
MET	P08581	1
PTPRF	P10586	1
VAPA	Q9P0L0	1
ADGRG6	Q86SQ4	3
BSG	P35613	2
CA2	P00918	3
CD44	P16070	2
CD46	P15529	2
CDH11	P55287	2
CDH2	P19022	2
CDH6	P55285	999
CEMIP2	Q9UHN6	2
CSPG4	Q6UVK1	2
DAG1	Q14118	2
DSC3	Q14574	2
GPC1	P35052	2
HLA-A	P04439	1
HLA-B	P01889	2
HSPG2	P98160	2
ITGA3	P26006	2
ITGA6	P23229	2
ITGB1	P05556	2
LOXL2	Q9Y4K0	3
MICB	Q29980	999
NT5E	P21589	2
PLXNB2	O15031	2
PODXL	O00592	2
S100A10	P60903	2
SLC39A10	Q9ULF5	3

Supplementary Fig. 9. Biotinylation by EGFR-FabID in Gefitinib alone-treated A431 and NCI-H226 cells.

a, Volcano blot of peptides detected as biotinylated peptides by LC-MS/MS. Each treatment of A431 or NCI-H226 cells was biotinylated with EGFR-FabID in three or five independent 10cm dishes,

respectively. Cell lysates reacted in independent replicates were pooled. The pooled samples were divided into three samples, purified separately, and analysed by mass spectrometry. **b,** Table of extracellular proteins identified by mass spectrometry (EGFR-FabID/AGIA-FabID ratios > 1 and $P < 0.05$). Proteins in black represent those known to interact with EGFR, whereas those in blue represent new EGFR-interacting proteins.

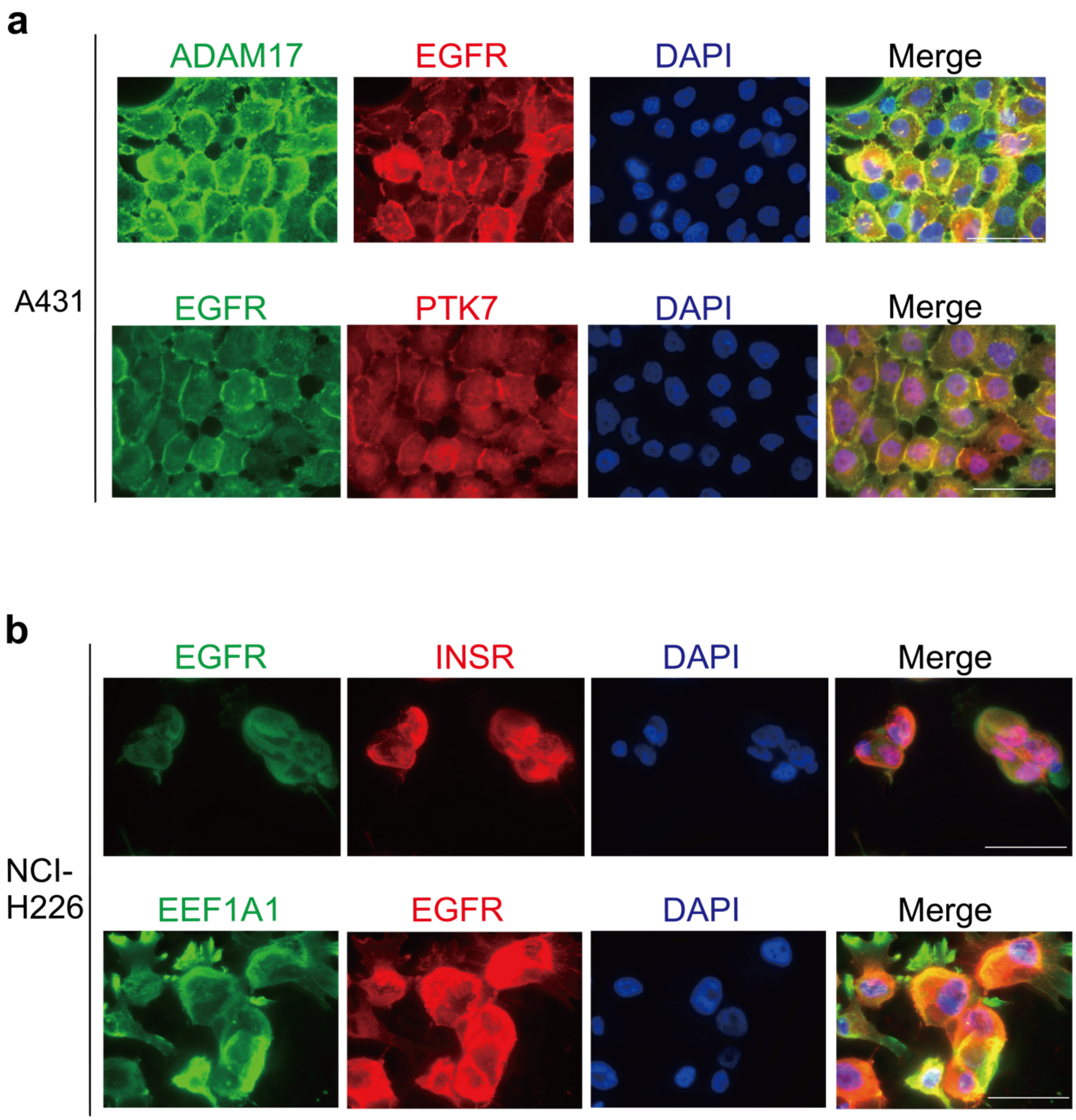

Supplementary Fig. 10. Immunostaining of ADAM17, PTK7, INSR, and EEF1A1 to confirm co-localisation with EGFR.

a, ADAM17 or PTK7 was co-localised with EGFR in A431 cells. Scale bar represents 50 μm. **b,** INSR or EEF1A1 colocalized with EGFR in NCI-H226 cells. Scale bar represents 50 μm.
