# Supplementary material for: Proximity extracellular protein-protein interaction analysis of EGFR using AirID-conjugated fragment of antigen binding
Source: Nat Commun. 2023 Dec 14;14:8301. doi: 10.1038/s41467-023-43931-7 (PMC10721602; doi:10.1038/s41467-023-43931-7)
Supplement: Supplementary file 3 — Description of Additional Supplementary Files [file 41467_2023_43931_MOESM3_ESM.pdf]

Description of Additional Supplementary Files:

Supplementary Data 1: Project description Expi293F cells were transfected with pcDNA3.1-EGFR and treated with AGIA-FabID or EGFR-FabID in three biological replicates. Cell lysates were subjected to methanol-chloroform precipitation and digested with trypsin. Biotinylated peptides were purified by Tamavidin 2-REV and identified by LC-MS/MS analysis.

Supplementary Data 2: Expi293F cells stably expressing EGFR were treated with AGIA-FabID or EGFR-FabID in three biological replicates. Cell lysates were subjected to methanol-chloroform precipitation and digested with trypsin. Biotinylated peptides were purified by Tamavidin 2-REV and identified by LC-MS/MS analysis.

Supplementary Data 3: A431 cells were pretreated with DMSO or gefitinib and unstimulated or stimulated with EGF. Then AGIA-FabID or EGFR-FabID were added to the cells in three biological replicates. Cell lysates were subjected to methanol-chloroform precipitation and digested with trypsin. Biotinylated peptides were purified by Tamavidin 2-REV and identified by LC-MS/MS analysis.

Supplementary Data 4: NCI-H226 cells were pretreated with DMSO or gefitinib and unstimulated or stimulated with EGF. Then AGIA-FabID or EGFR-FabID were added to the cells in three biological replicates. Cell lysates were subjected to methanol-chloroform precipitation and digested with trypsin. Biotinylated peptides were purified by Tamavidin 2-REV and identified by LC-MS/MS analysis.

Supplementary Data 5: Protein data for Supplementary Table 3

Supplementary Data 6: Protein data for Supplementary Table 4.

Supplementary Data 7: A431cells were pretreated with DMSO or gefitinib. Then AGIA-FabID or EGFR-FabID were added to the cells in three biological replicates. Cell lysates were subjected to methanol-chloroform precipitation and digested with trypsin. Biotinylated peptides were purified by Tamavidin 2-REV and identified by LC-MS/MS analysis.

Supplementary Data 8: NCI-H226 cells were pretreated with DMSO or gefitinib. Then AGIA-FabID or EGFR-FabID were added to the cells in three biological replicates. Cell lysates were subjected to methanol-chloroform precipitation and digested with trypsin. Biotinylated peptides were purified by Tamavidin 2-REV and identified by LC-MS/MS analysis.
