# Supplementary material for: Proximity extracellular protein-protein interaction analysis of EGFR using AirID-conjugated fragment of antigen binding
Source: Nat Commun. 2023 Dec 14;14:8301. doi: 10.1038/s41467-023-43931-7 (PMC10721602; doi:10.1038/s41467-023-43931-7)
Supplement: Supplementary file 12 — Reporting Summary [file 41467_2023_43931_MOESM12_ESM.pdf]

nature portfolio

Corresponding author(s): Hidetaka Kosako,Tatsuya Sawasaki

Last updated by author(s): 2023/12/6

Reporting Summary

Nature Portfolio wishes to improve the reproducibility of the work that we publish. This form provides structure for consistency and transparency in reporting. For further information on Nature Portfolio policies, see our Editorial Policies and the Editorial Policy Checklist.

Statistics

For all statistical analyses, confirm that the following items are present in the figure legend, table legend, main text, or Methods section.

n/a	Confirmed	
☐	☒	The exact sample size (*n*) for each experimental group/condition, given as a discrete number and unit of measurement
☐	☒	A statement on whether measurements were taken from distinct samples or whether the same sample was measured repeatedly
☐	☒	The statistical test(s) used AND whether they are one- or two-sided<br>*Only common tests should be described solely by name; describe more complex techniques in the Methods section.*
☒	☐	A description of all covariates tested
☐	☒	A description of any assumptions or corrections, such as tests of normality and adjustment for multiple comparisons
☐	☒	A full description of the statistical parameters including central tendency (e.g. means) or other basic estimates (e.g. regression coefficient) AND variation (e.g. standard deviation) or associated estimates of uncertainty (e.g. confidence intervals)
☐	☒	For null hypothesis testing, the test statistic (e.g. *F*, *t*, *r*) with confidence intervals, effect sizes, degrees of freedom and *P* value noted<br>*Give P values as exact values whenever suitable.*
☒	☐	For Bayesian analysis, information on the choice of priors and Markov chain Monte Carlo settings
☒	☐	For hierarchical and complex designs, identification of the appropriate level for tests and full reporting of outcomes
☒	☐	Estimates of effect sizes (e.g. Cohen's *d*, Pearson's *r*), indicating how they were calculated

Our web collection on statistics for biologists contains articles on many of the points above.

Software and code

Policy information about availability of computer code

Data collection	All data collection in this study were performed using softwares attached to each detector.<br>Image Quant LAS 4000 software (GE Healthcare, version 1.1) for chemical luminescent immunoblot.<br>Wallac Envision Manager software (ParkinElmer, version 1.12) for AlphaScreen.<br>SpectraMax ID3 software (Molecular Device, version 7.1) for MTS assay.<br>BZ-X800 viewer Software (KEYENCE, version 1.2.3) for Immunofluorescent.<br>Proteome Discoverer software (Thermo Fisher Scientific, version 2.5) for MS proteomics data analysis.<br>Ahpafold2 (ColabFold, version 1.5.3) for protein structure prediction.<br>Drug Target Excavator (DTX) (in house, version 1.0b) for PPI analysis
Data analysis	Image analysis was performed using Fiji software (version 2.9.0) or ImageJ (version 2.0.0).<br>FlowJo software (Tree Star, Ashland, OR, USA, version 10.8.1) for Flow cytometry.<br>BZ-X800 Analyzer (KEYENCE, version 1.1.21)for Immunofluorescent.<br>Data analysis and significant changes were performed using Microsoft Excel (version 16.78.3) or GraphPad Prism 9 (version 9.4.1).<br>Cytoscape (version 3.9.1) for drawing PPI graphs.<br>Pymol (Schrödinger, version 2.5) for molecule visualization and structure analysis.

For manuscripts utilizing custom algorithms or software that are central to the research but not yet described in published literature, software must be made available to editors and reviewers. We strongly encourage code deposition in a community repository (e.g. GitHub). See the Nature Portfolio guidelines for submitting code & software for further information.

Data

Policy information about availability of data

All manuscripts must include a data availability statement. This statement should provide the following information, where applicable:

- Accession codes, unique identifiers, or web links for publicly available datasets
- A description of any restrictions on data availability
- For clinical datasets or third party data, please ensure that the statement adheres to our policy

All data analysed in this study are included in this published article and supplementary information files. All data involved in this study are available by reasonable request to the corresponding author.The MS proteomics data have been provided in Supplementary Table 1–8 and deposited to the ProteomeXchange Consortium via the jPOST partner repository with the dataset identifiers PXD039449 [http://proteomecentral.proteomexchange.org/cgi/GetDataset?ID=PXD039449] (Proximity biotinylation of Expi293F cells overexpressing EGFR by EGFR-FabID),PXD043525 [http://proteomecentral.proteomexchange.org/cgi/GetDataset?ID=PXD043525] (Proximity biotinylation of Expi293F cells stably expressing EGFR by EGFR-FabID), PXD039450 [http://proteomecentral.proteomexchange.org/cgi/GetDataset?ID=PXD039450] (Proximity biotinylation of A431 cells by EGFR-FabID), PXD039451 [http://proteomecentral.proteomexchange.org/cgi/GetDataset?ID=PXD039451] (Proximity biotinylation of NCI-H226 cells by EGFR-FabID), PXD043526 [http://proteomecentral.proteomexchange.org/cgi/GetDataset?ID=PXD043526] (Proximity biotinylation of A431 cells in the gefitinib alone treatment by EGFR-FabID), and PXD043527 [http://proteomecentral.proteomexchange.org/cgi/GetDataset?ID=PXD04527] (Proximity biotinylation of NCI-H226 cells in the gefitinib alone treatment by EGFR-FabID).

Research involving human participants, their data, or biological material

Policy information about studies with human participants or human data. See also policy information about sex, gender (identity/presentation), and sexual orientation and race, ethnicity and racism.

Reporting on sex and gender	N\A
Reporting on race, ethnicity, or other socially relevant groupings	N\A
Population characteristics	N\A
Recruitment	N\A
Ethics oversight	N\A

Note that full information on the approval of the study protocol must also be provided in the manuscript.

Field-specific reporting

Please select the one below that is the best fit for your research. If you are not sure, read the appropriate sections before making your selection.

[x] Life sciences [ ] Behavioural & social sciences [ ] Ecological, evolutionary & environmental sciences

For a reference copy of the document with all sections, see nature.com/documents/nr-reporting-summary-flat.pdf

Life sciences study design

All studies must disclose on these points even when the disclosure is negative.

Sample size	We have chosen three independent experiments (n = 3) as a minimum size for statistics. For immunoblot analyses and LC-MS/MS analysis, such as biotinylation by EGFR-FabID and streptavidin pull-down assays, we have chosen more than two independent according to our previous experience in same research field (25. Yamanaka, S. et al., A proximity biotinylation-based approach to identify protein-E3 ligase interactions induced by PROTACs and molecular glues. Nat. Commun. 13, 183 (2022).) and traditional experimental approach in biochemical and cellular experiments.
Data exclusions	No data were excluded from the analyses.
Replication	In mass spectrometry experiments, replication was performed as indicated in the methods section or figure legend.<br>Numbers of replicates are described in the figure legends.
Randomization	Randomization was not relevant because there is no allocation of samples/organisms/participants involved in this study.
Blinding	Binding was not necessary because there is no group allocation involved in this study.

Reporting for specific materials, systems and methods

We require information from authors about some types of materials, experimental systems and methods used in many studies. Here, indicate whether each material, system or method listed is relevant to your study. If you are not sure if a list item applies to your research, read the appropriate section before selecting a response.

Materials & experimental systems

n/a	Involved in the study
☐	☒ Antibodies
☐	☒ Eukaryotic cell lines
☒	☐ Palaeontology and archaeology
☒	☐ Animals and other organisms
☒	☐ Clinical data
☒	☐ Dual use research of concern
☒	☐ Plants

Methods

n/a	Involved in the study
☒	☐ ChIP-seq
☐	☒ Flow cytometry
☒	☐ MRI-based neuroimaging

Antibodies

Antibodies used

The following horseradish peroxidase (HRP)-conjugated antibodies were used in this study: anti-FLAG (#A8592,Sigma-Aldrich, WB 1:5000, RRID:AB_439702), anti-AGIA (produced in our laboratory, WB 1:10000)28, anti-tubulin (#PM054-7, MBL, WB 1:5000, RRID:AB_10695326), anti-His (#sc-8036, Santa Cruz, WB 1:1000, RRID:AB_627727), and biotin (#7075, Cell Signalling Technology, WB 1:1000, RRID:AB_10696897). The following primary antibodies were used: anti-EGFR (clone EMab-134, WB 1:1000, PLA 1:100, IF 1:100,)34,35, anti-EGFR (#4267, Cell Signalling Technology, IF 1:100, RRID:AB_2864406), anti-ADAM17 (#3976, Cell Signalling Technology, IF 1:100, PLA 1:100, RRID:AB_2242380), anti-ADAM17 (#sc-390859, SANTA CRUZ, IF 1:100), anti-P-EGFR-Tyr1173 (#4407, Cell Signalling Technology, WB 1:1000, RRID:AB_331795), anti-biotin (#5597, Cell Signalling Technology, WB 1:1000, IF 1:100, RRID:AB_10828011), anti-insulin receptor alpha (#74118, Cell Signalling Technology, PLA 1:100, RRID:AB_2799850), anti-INSR (#A19067, ABclonal, IF 1:100, RRID:AB_2862559), anti-STAT3 (#9132, Cell Signalling Technology, WB 1:1000, RRID:AB_331588), anti-P-STAT3-Y705 (#9145, Cell Signalling Technology, WB 1:1000, RRID:AB_2491009), anti-EEF1A1 (#sc-21758, SANTA CRUZ, IF 1:50, RRID:AB_309663) and anti-PTK7 (#17799-1-AP, proteintech, IF 1:100, PLA 1:100, RRID:AB_2878442). Anti-rabbit IgG (#7074, HRP-conjugated, Cell Signalling Technology, WB 1:10000, RRID:AB_2099233), anti-mouse IgG (#7076, HRP-conjugated, Cell Signalling Technology, WB 1:10000, RRID:AB_330924), F(ab')2-Goat anti-Rabbit IgG (H+L) Cross-Adsorbed Secondary Antibody, Alexa Fluor 555 (#A21431, Thermo Fisher Scientific, IF 1:1000, RRID:AB_2535852), and goat anti-mouse IgG (H+L) Cross-Adsorbed Secondary Antibody, Alexa Fluor™ 488 (#A11001, Thermo Fisher Scientific, IF 1:1000, RRID:AB_2534069) were used as secondary antibodies.

Validation

All primary antibodies in this study were purchased from commercial companies. All of these antibodies were stated to be able to detect each endogenous protein in supplier's datasheets and these antibodies were used according to supplier's protocol.
The anti-ADAM17 rabbit pAb (Cell Signalling Technology, #3976, 1:1000), anti-P-EGFR-Tyr1173 rabbit mAb (Cell Signalling Technology, #4407, 1:1000), anti-biotin rabbit mAb (Cell Signalling Technology, #5597, 1:1000), anti-insulin receptor alpha rabbit mAb (Cell Signalling Technology, #74118, 1:1000), anti-STAT3 rabbit pAb (Cell Signalling Technology, #9132, 1:1000), anti-P-STAT3-Y705 rabbit mAb (Cell Signalling Technology, #9145, 1:1000) have been validated for detection of human species by immunoblot analysis as described on Cell SignalingTechnology websites for specific antibodies.
The anti-ADAM17 (#sc-390859, SANTA CRUZ, IF 1:100), anti-EEF1A1 (#sc-21758, SANTA CRUZ, IF 1:50, RRID:AB_309663) and anti-EGFR (#4267, Cell Signalling Technology, IF 1:100, RRID:AB_2864406) have been validated for detection of human species by immunostaining analysis as described on SANTA CRUZ or Cell SignalingTechnology websites for specific antibodies.
anti-INSR (# A19067,ABclonal ,IF 1:100 RRID:AB_2862559) have been validated for detection of human species as described on ABclonal websites for specific antibodies (https://abclonal.co.jp/index.php?s=/catalog-antibodies/InsulinReceptorRabbitmAb/A19067).
The anti-PTK7 rabbit pAb (proteintech, 17799-1-AP, 1:1000) has been validated for detection of human species by mmunoblot analysis as described on proteintech websites for specific antibodies.
The anti-FLAG mAb(HRP-conjugated, Sigma-Aldrich, A8592, 1:5000), anti-His (Santa Cruz, sc-8036, 1:1000) have been validated for detection of epitope-tagged proteins by immunoblot analysis as described on each supplier's website for specific antibodies.
The anti-α-tubulin rabbit pAb (HRP-conjugated, MBL, # PM054-7, 1:5000) have been validated for detection of human α-tubulin by immunoblot analysis as described on each supplier's website.
The AGIA antibody has been validated in published paper (Yano, T. et al., PLoS ONE 11: e0156716). cited in Methods. Emab-134 antibody has been validated in published paper (Kaneko, M.K. et al., Biochem Biophys Rep 14, 54-57). cited in Methods.

Eukaryotic cell lines

Policy information about cell lines and Sex and Gender in Research

Cell line source(s)

Expi293F cell line (RRID:CVCL_D615) was purchased from Gibco/Thermo Fisher Scientific. A431 (JCRB9009)(RRID:CVCL_0037) and HeLa-S3 (JCRB9010)(RRID:CVCL_0058) cell line was purchased from Japanese Collection of Research Bioresources Cell Bank (JCRB Cell Bank). NCI-H1975(RRID:CVCL_1511) and NCI-H226 call lines(RRID:CVCL_1544) were purchased from American Type Culture Collection (ATCC).

Authentication

All cell lines were used for each experiment between passage number 5 and 15 to avoid changes in the cell line's properties. In addition, all cell lines were authenticated by morphological appearances by careful observation.

Mycoplasma contamination

All cell lines used in this study were tested negative for mycoplasma.

Commonly misidentified lines (See ICLAC register)	No commonly misidentified cell lines were used in this sturdy.

Plants

Seed stocks	N/A
Novel plant genotypes	N/A
Authentication	N/A

Flow Cytometry

Plots

Confirm that:

- [x] The axis labels state the marker and fluorochrome used (e.g. CD4-FITC).
- [x] The axis scales are clearly visible. Include numbers along axes only for bottom left plot of group (a 'group' is an analysis of identical markers).
- [x] All plots are contour plots with outliers or pseudocolor plots.
- [x] A numerical value for number of cells or percentage (with statistics) is provided.

Methodology

Sample preparation	Sample preparation listed in Methods
Instrument	BD FACSLyricTM Flow Cytometer (BD Biosciences)
Software	Data were collected with BD FACSuite software (BD Biosciences), and the established files were subsequently analyzed using FlowJo software (Tomy Digital Biology).
Cell population abundance	Approximately 30,000 cellular events were collected and the fluorescence intensity of biotinilated cells population by EGFR-FabID was calculated.
Gating strategy	Dead cells were excluded based on FSC-A and SSC-A characteristics.

- [ ] Tick this box to confirm that a figure exemplifying the gating strategy is provided in the Supplementary Information.
